# Oxidative Stress and Heart Failure: Mechanisms, Signalling Pathways, and Therapeutics

**DOI:** 10.1155/2022/9829505

**Published:** 2022-04-14

**Authors:** Luana Urbano Pagan, Mariana Janini Gomes, Paula Felippe Martinez, Marina Politi Okoshi

**Affiliations:** ^1^Botucatu Medical School, Sao Paulo State University, UNESP, Botucatu, Brazil; ^2^Brigham and Women's Hospital, Harvard Medical School, Boston, USA; ^3^Integrated Institute of Health, Federal University of Mato Grosso do Sul, UFMS, Campo Grande, Brazil

Heart failure is an important public health issue due to its poor prognosis and high prevalence, morbidity, and mortality [1]. Heart failure is clinically characterized by a reduced capacity for physical exercise and daily activities as a result of the early occurrence of fatigue and dyspnea.

Oxidative stress, defined as an imbalance between oxygen radical production and scavenging, plays an important role in the pathophysiology of cardiac remodeling and heart failure [2, 3]. Clinical and experimental studies have provided substantial evidences that oxidative stress is increased in the myocardium and at a systemic level during heart failure [4, 5]. Although at physiological levels, reactive oxygen species (ROS) play important roles in intracellular pathways and redox signaling, they may induce cellular dysfunction and damage at higher levels. Despite extensive investigation, the molecular pathways involved in heart failure-associated oxidative stress are still not completely understood.

Common causes of heart failure include myocardial infarction, systemic arterial hypertension, valve disease, and cardiomyopathy [6]. This Special Issue consists of nine original articles that investigated cellular and molecular processes involved in the oxidative stress associated with heart failure. The manuscripts approached distinctive causes of heart failure in both humans and animals, improving our understanding of signaling pathways and mechanisms of novel targets for heart failure prevention and therapy. In this Editorial, we provide an overview of these articles highlighting the major finds of each one.

Several animal models that mimic heart failure have been used to gain insight into the complex biology of this disease [7–9]. Three studies included in this Special Issue investigated cardiotoxicity-induced heart failure caused by chemotherapy drugs widely used in cancer treatment. Zhang et al. [10] evaluated by peptidomics changes in peptide profiles related to doxorubicin- (DOX-) induced cardiotoxicity and successfully identified differentially expressed peptides in mouse cardiac tissue. Through bioinformatics analyses, the authors identified a candidate peptide for protecting the myocardium against DOX-induced cell apoptosis, thus providing a new approach for the treatment of DOX-induced cardiotoxicity. Modesto et al. [11] reinforced the oxidative stress role on the mechanisms involved in DOX-induced cardiotoxicity in rats. DOX leads to lipid peroxidation and lowered activity of antioxidant enzymes, which were combined with inflammation, energy metabolism changes, and cytotoxicity. Similarly, Gholami et al. [12] also demonstrated evidence to support the involvement of oxidative stress in the pathogenesis of cardiotoxicity induced by another chemotherapy drug, the arsenic trioxide. At least in part, green tea attenuated oxidative stress and cytotoxic damage. The authors revealed a link between the antioxidant effects of pentoxifylline and its therapeutic potential against cardiac oxidative damage.

Besides cardiotoxicity, this Special Issue presents manuscripts that investigated pressure overload-induced cardiac remodeling in different animal models. Horvath et al. [13] investigated the cardioprotective effect of BGP-15, an insulin signaling-related molecule, in an animal model of hypertension-induced heart failure. Their major findings include a BGP-15 positive effect on cardiac function and the remodeling process by inhibition of profibrotic signaling factors and promotion of mitochondrial biogenesis. Zhang et al. [14] used a mouse model of pathological cardiac hypertrophy caused by transverse aortic constriction. The authors verified that the newfound proliferator-activated receptor-gamma coactivator (PGC)-1*α*/activating transcription factor 5 (ATF5) axis can partly activate mitochondrial unfolding protein response and mediate the protective role of tetrahydrocurcumin against pressure overload-induced cardiac hypertrophy and oxidative stress. The results demonstrate a possible therapeutic action of tetrahydrocurcumin in heart failure caused by pressure overload. Also in the transverse aortic constriction model, Peng et al. [15] studied the effects of oxidative stress in inducing heart failure and unraveled a specific action mechanism underlying the role of LCZ696, a drug recommended for the treatment of heart failure with reduced ejection fraction. The authors showed that Sirt3 may be a therapeutic target in the heart failure treatment, as the cardioprotective effects of LCZ696 were partly mediated by the Sirt3-dependent pathway.

Considering inflammation associated with oxidative stress plays a role in the pathophysiology of many chronic diseases [16], including heart failure, animal models of sepsis have been often used to investigate molecular mechanisms involved in cardiac injury. Interleukin- (IL-) 16 is an important inflammatory mediator and a potential pharmacologic target in heart failure. The study by Zhang et al. [17] evaluated whether IL-16 participates in sepsis-induced cardiac injury and dysfunction in mice through the regulation of oxidative stress. The study demonstrated that IL-16 neutralization may positively regulate the Nrf2 pathway, reduce oxidative stress, and inhibit the transfer of mitochondrial apoptosis-inducing factor from mitochondria to the nucleus, and thus reduce cardiomyocyte apoptosis and myocardial injury and improve cardiac function in sepsis rats.

Pharmacological treatment recommended by guidelines for heart failure has progressed over the past decades and has improved the patient prognosis [18]. However, the importance of tailoring the treatment has been emphasized as different groups of patients benefit more from specific therapies. Wojciechowska et al. [19] analyzed the influence of the redox balance parameters on the prognosis of 707 patients with heart failure with reduced ejection fraction, taking into account ischemic and nonischemic etiology. The authors showed an association between different oxidative biomarkers in the heart failure progression depending on its etiology, therefore strengthening the importance of personalizing the heart failure treatment.

Beyond pharmacological treatment, physical exercise has been recommended as a nonpharmacological therapy for heart failure. Batista et al. [20] analyzed the impact of different modalities and intensities of exercise training on cardiac remodeling started early after experimental myocardial infarction. The authors showed that both high-intensity interval and continuous low-intensity modalities improved cardiac energetic metabolism in comparison with control infarcted rats. In addition, high-intensity interval training decreased cardiac oxidative stress, which was associated with improved diastolic function.

We hope that this Special Issue has provided new insights into the pathways and mechanisms involved in oxidative stress associated with heart failure and stimulated new research ideas and collaborations that can benefit advances in the heart failure treatment.

## References

[1] Tsao C. W., Aday A. W., Almarzooq Z. I. (2022). Heart disease and stroke statistics-2022 update: a report from the American Heart Association. *Circulation*.

[2] Tsutsui H., Kinugawa S., Matsushima S. (2011). Oxidative stress and heart failure. *American Journal of Physiology. Heart and Circulatory Physiology*.

[3] Pagan L. U., Gomes M. J., Gatto M., Mota G. A. F., Okoshi K., Okoshi M. P. (2022). The role of oxidative stress in the aging heart. *Antioxidants*.

[4] Martinez P. F., Bonomo C., Guizoni D. M. (2016). Modulation of MAPK and NF-kappaB signaling pathways by antioxidant therapy in skeletal muscle of heart failure rats. *Cellular Physiology and Biochemistry*.

[5] Rosa C. M., Gimenes R., Campos D. H. (2016). Apocynin influence on oxidative stress and cardiac remodeling of spontaneously hypertensive rats with diabetes mellitus. *Cardiovascular Diabetology*.

[6] Schwinger R. H. G. (2021). Pathophysiology of heart failure. *Cardiovascular Pathology*.

[7] Lima A. R. R., Martinez P. F., Damatto R. L. (2014). Heart failure-induced diaphragm myopathy. *Cellular Physiology and Biochemistry*.

[8] Guimaraes J. F., Muzio B. P., Rosa C. M. (2015). Rutin administration attenuates myocardial dysfunction in diabetic rats. *Cardiovascular Diabetology*.

[9] Martinez P. F., Bonomo C., Guizoni D. M. (2015). Influence of N-acetylcysteine on oxidative stress in slow-twitch soleus muscle of heart failure rats. *Cellular Physiology and Biochemistry*.

[10] Zhang L., Wang X., Feng M. (2020). Peptidomics analysis reveals peptide pdcryab1 inhibits doxorubicin-induced cardiotoxicity. *Oxidative Medicine and Cellular Longevity*.

[11] Modesto P. N., Polegato B. F., Dos Santos P. P. (2021). Green tea (Camellia sinensis) extract increased topoisomerase II *β*, improved antioxidant defense, and attenuated cardiac remodeling in an acute doxorubicin toxicity model. *Oxidative Medicine and Cellular Longevity*.

[12] Gholami A., Ataei S., Ahmadimoghaddam D., Omidifar N., Nili-Ahmadabadi A. (2021). Pentoxifylline attenuates arsenic trioxide-induced cardiac oxidative damage in mice. *Oxidative Medicine and Cellular Longevity*.

[13] Horvath O., Ordog K., Bruszt K. (2021). BGP-15 protects against heart failure by enhanced mitochondrial biogenesis and decreased fibrotic remodelling in spontaneously hypertensive rats. *Oxidative Medicine and Cellular Longevity*.

[14] Zhang B., Tan Y., Zhang Z. (2020). Novel PGC-1 *α*/ATF5 axis partly activates UPR mt and mediates cardioprotective role of tetrahydrocurcumin in pathological cardiac hypertrophy. *Oxidative Medicine and Cellular Longevity*.

[15] Peng S., Lu X. F., Qi Y. D. (2020). LCZ696 ameliorates oxidative stress and pressure overload-induced pathological cardiac remodeling by regulating the Sirt3/mnSOD pathway. *Oxidative Medicine and Cellular Longevity*.

[16] Ranneh Y., Ali F., Akim A. M., Hamid H. A., Khazaai H., Fadel A. (2017). Crosstalk between reactive oxygen species and pro-inflammatory markers in developing various chronic diseases: a review. *Applied Biological Chemistry*.

[17] Zhang J., Yang Z., Liang Z. (2021). Anti-interleukin-16 neutralizing antibody treatment alleviates sepsis-induced cardiac injury and dysfunction via the nuclear factor erythroid-2 related factor 2 pathway in mice. *Oxidative Medicine and Cellular Longevity*.

[18] McDonagh T. A., Metra M., Adamo M. (2021). 2021 ESC guidelines for the diagnosis and treatment of acute and chronic heart failure. *European Heart Journal*.

[19] Wojciechowska C., Jacheć W., Romuk E. (2021). Serum sulfhydryl groups, malondialdehyde, uric acid, and bilirubin as predictors of adverse outcome in heart failure patients due to ischemic or nonischemic cardiomyopathy. *Oxidative Medicine and Cellular Longevity*.

[20] Batista D. F., Polegato B. F., Da Silva R. C. (2020). Impact of modality and intensity of early exercise training on ventricular remodeling after myocardial infarction. *Oxidative Medicine and Cellular Longevity*.

